# Identification of soybean peptide leginsulin variants in different cultivars and their insulin-like activities

**DOI:** 10.1038/s41598-018-35331-5

**Published:** 2018-11-15

**Authors:** Tsutomu Hashidume, Taiken Sakano, Ayaka Mochizuki, Keisuke Ito, Sohei Ito, Yasuaki Kawarasaki, Noriyuki Miyoshi

**Affiliations:** 1 Graduate School of Integrated Pharmaceutical & Nutritional Sciences, University of Shizuoka, Shizuoka, 422-8526 Japan; 2 School of Food & Nutritional Sciences, University of Shizuoka, Shizuoka, 422-8526 Japan

## Abstract

We have recently reported that green soybean cultivar, *echigomidori*, and not the yellow cultivar, *fukuyutaka*, is a rich source of hormone-like peptide leginsulin consisting of 37 amino acids (Leg_1_37, PDB 1JU8A) and its C-terminal glycine deletant, Leg_1_36. Green soybean is mature, but the color of the seedcoat and cotyledon remains green. Therefore, in this study, we examined the leginsulin content in different varieties of 11 colored soybeans (including green, yellow, red, brown and black) and *edamame* (immature soybean). Profile analysis of soybean constituents by LC-MS showed that Leg_1 (36 + 37) detected as a prominent peak in 3 green and 1 yellow soybean cultivar was the strongest contributor in principal component analysis, indicating Leg_1 is the most characteristic feature for distinguishing soybean cultivars. However, smaller amounts of leginsulin-like peptides, defined as Leg_2 and Leg_3, were detected in other samples. The cDNA sequences and LC-MS/MS analyses revealed that Leg_2 was a homologue of Leg_1 with three amino acid substitutions derived from SNPs, while Leg_3 was a Leg_1/Leg_2 paralog. Expression levels of Leg_1 were markedly higher than Leg_2 and Leg_3. Additionally, in glucose uptake assay, purified TRX-His-tag fused recombinant Leg_1_37 prepared by bacterial expression showed stronger insulin-like activities than other variants including Leg_2, Leg_3, and their Gly deletants in myotube-like differentiated L6 and C2C12 cells. These results suggest that dietary consumption of soybean seed, especially including a higher amount of Leg_1_37, could be useful for lowering of blood glucose.

## Introduction

**Figure 1:**
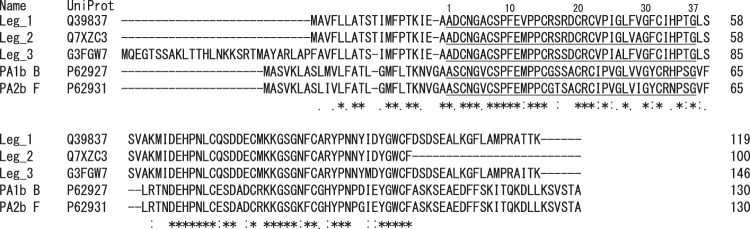
Sequence alignment of leginsulin and PA1b and their precursor proteins. Peptide sequences for leginsulin (Leg_1–3) and PA1b B and F are underlined. Note that Leg_2 and Leg_3 were first found in this study.

Leginsulin (PDB 1JU8A), a peptide consisting of 37 amino acids (leginsulin variant 1 consists of 37 amino acids; Leg_1_37) and its C-terminal glycine deletion form (Leg_1_36), have been found in radicles of germinated soybean (*Glycine max*) seeds as a high affinity molecule for 43 kDa basic 7 S globulin protein (Bg)^1^. Leg_1_37 and Leg_1_36 are formed by the post-translational processing of a precursor protein, soybean albumin 1 (UniProt entry Q39837, sequence in Fig. 1). Pea albumin 1 (PA1) is the homologue protein (UniProt entry P62926-P62931), which is also processed to produce PA1a and PA1b peptides. Several leginsulin homologues including the PA1b family (e.g. PA1b A - F) have been identified. These peptides are sometimes inclusively called leginsulin or aglycin in some literature^2–4^. Leginsulin receptor Bg has a protein kinase domain and is stimulated by Leg_1_37 resulted in the induction of self-phosphorylation^1^. Bg activation is considered important for signal transduction associated with cell growth and differentiation in plant cells^5^. However, Bg phosphorylation was not induced when three intramolecular disulfide bonds of Leg_1_37 were treated with reducing reagent^1^. This finding indicates that the peptide structure of Leg_1_37 consists of three intramolecular disulfide bonds and is important in the ligand activity. Interestingly, mammalian insulin and insulin-like growth factor are also capable of binding to leginsulin receptor Bg, although they have no peptide sequence similarities with leginsulin^6,7^. Moreover leginsulin and PA1 peptides show resistance to heat and several digestion enzymes^3,8^. It is therefore suggested that leginsulin could be a potential insulin-like peptide hormone not only in plant but also animal bodies. However, recent animal experiments have shown conflicting results. Dun *et al*. demonstrated that a single subcutaneous injection of a leginsulin homologue PA1bB (10 µg/g body weight) to fasted mice significantly increased blood glucose concentration^3^. The sequence identity of PA1bB with Leg_1 is 68% (Fig. 1). In contrast, daily oral administration of PA1bB (called aglycin in their study) inhibited the streptozotocin and high fat diet-induced hyperglycemia and ameliorated insulin resistance in BALB/c mice^2^. In addition, one of the homologous peptide, PA1bF, sharing 59% sequence identity with Leg_1 (Fig. 1), which shows strong insecticidal activity in several insect pests^9,10^. These findings indicate that the discriminative bioactivities of leginsulin and its homologue would be rather depending on the variation of their amino acid sequences.

**Figure 2:**
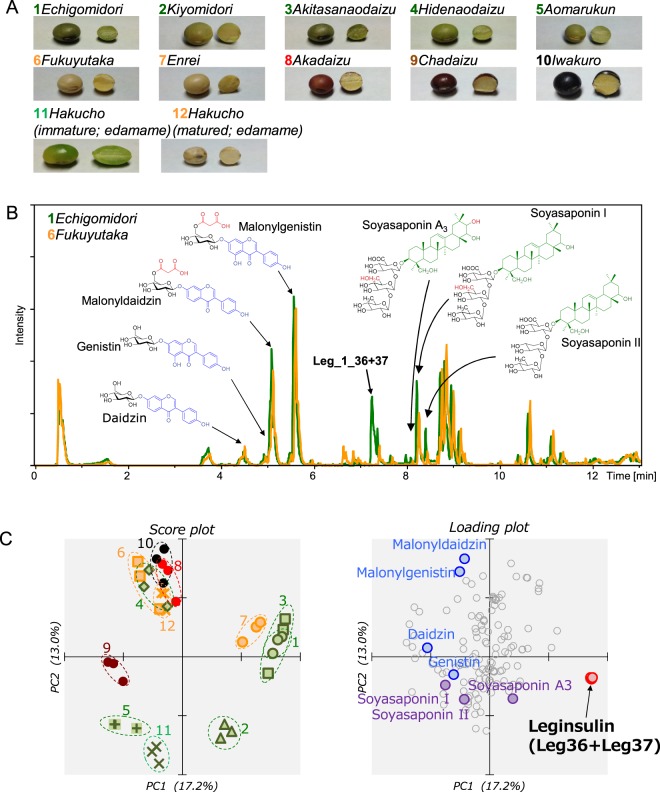
LC-MS analysis of soybean extract. (**A**) Appearance of 12 soybean samples used in this study. (**B**) Typical base peak ion chromatograms obtained by analysis of *echigomidori* (1, green) and *fukuyutaka* (6, yellow). Leginsulin (Leg_1_36 + 37) and major isoflavones and saponins detected are indicated by arrows. (**C**) Profile of soybean constituents. Score plot and loading plot of PCA.

In our previous work, elevated levels of Leg_1_37 and Leg_1_36 was detected in green-colored soybean cultivar *echigomidori* but not in yellow soybean cultivar *fukuyutaka*^11^. Green soybean is mature soybean seed, but the color of the seedcoat and cotyledon is still green, so it is not same as immature soybean, *edamame* (Fig. 2A). Therefore in this study we examined leginsulin contents in several soybean cultivars including green, yellow, red, brown and black-colored soybean. The leginsulin contents in mature and immature *edamame* seed were also evaluated. We detected and identified three homologous leginsulins, Leg_1, Leg_2 and Leg_3 by LC-MS and the cDNA sequencing analyses of these samples. We found that Leg_1 is a highly expressed variant and co-producing C-terminal glycine deletant. Six recombinant leginsulin (36 or 37 amino acid peptide of Leg_1~3, respectively) were prepared in this study, which showed insulin-like activity in myotube-like differentiated rat L6 and mouse C2C12 cells.

## Results

### LC-MS analyses of EtOH extract of soybeans

We have previously performed LC-MS analysis of green soybean (cultivar *echigomidori*) seed extract to detect leginsulin, which consists of 37 amino acids and corresponds to PDB 1JU8A (Leg_1_37) and its C-terminal glycine deletant (Leg_1_36)^11^. Here we performed LC-MS analysis to examine the levels of leginsulin in 11 soybeans cultivars (12 samples, shown in Fig. 2A) and also to evaluate levels of other constituents including phytochemicals. Delipidated soybean samples were extracted by 70% EtOH, and then subjected to LC-MS. As shown in Fig. 2B, one of the prominent peaks eluted at 7.2 min was Leg_1 (Leg_1_36 and Leg_1_37), which was detected in *echigomidori*, but they were under the detection limit in *fukuyutaka*. Isoflavones including daidzin, genistin, malonyldaidzin and malonylgenistin, and saponins including soyasaponin A_3_, I and II were detected in all samples. Then, MS data were subjected to principle component analysis (PCA). The loading plot of PCA indicated that leginsulin (Leg_1_36 + 37) was the strongest contributor to the first principle component (X-axis), therefore indicating that Leg_1 is the strongest characteristic feature for distinguishing soybean cultivars (Fig. 2C). Therefore, the score plot of PCA data suggests that sample# 1, 2, 3, and 7 were expressing high levels of Leg_1 (Fig. 2C). Additionally, the most influential variable in second principle component (Y-axis) was malonyldaidzin.

**Figure 3:**
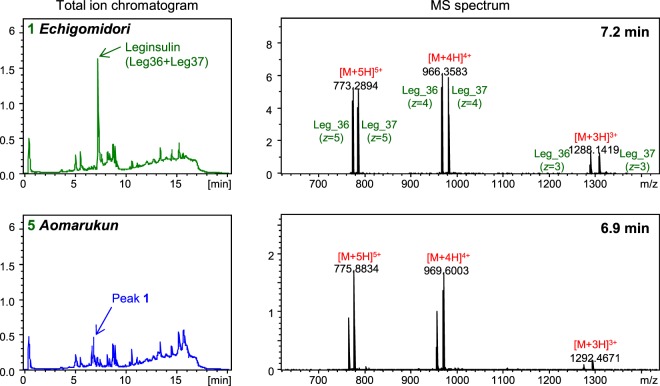
Total ion chromatogram and MS spectra of LC-MS analysis for *echigomidori* and *aomarukun*. MS spectra observed in *echigomidori* and *aomarukun* at 7.2 and 6.9 min for Leg_1 and Leg_1-like peptides, respectively, are shown.

Leg_1_36 and Leg_1_37 were not separated in the HPLC condition using reverse-phase column, and therefore were detected as paired ions (36 + 37) with multiple charges (*z* = 3~5, Fig. 3). Interestingly, a peak 1 eluted at 6.9 min was detected in *aomarukun* (sample# 5), of which MS spectrum shows an analogous pattern to that of Leg_1 (Fig. 3). The molecular mass difference of corresponding MS ions between Leg_1 (36 and 37) and those of *aomarukun* (sample# 5) was 44.047 Da. These results imply that *aomarukun* is probably producing leginsulin variants produced in place of Leg_1.

### LC-MS and cDNA sequence of leginsulin variants

**Figure 4:**
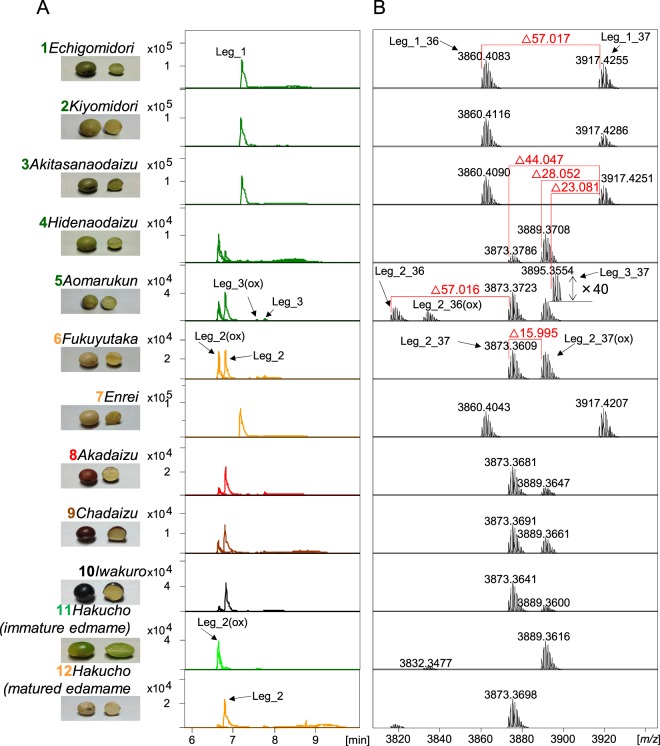
Extracted ion chromatograms and MS spectra for leginsulin and leginsulin variants in 11 soybeans cultivars (12 samples). (**A**) Extracted ion chromatograms for Leg_1 (*m/z* 965.9 and 980.2, *z* = 4, for Leg_1_36 and 37, respectively), Leg_2 (*m/z* 954.9 and 969.2, *z* = 4), Leg_2(ox) (*m/z* 958.9 and 973.2, *z* = 4), Leg_3 (*m/z* 960.2 and 974.4, *z* = 4), Leg_3(ox) (*m/z* 964.2 and 978.4, *z* = 4). Leginsulins were detected in all samples as shown by compound names and arrows in each sample. (**B**) Charge deconvoluted MS spectra (*z* = 1) of leginsulins. ∆57.017 or 57.016 Da correspond to Gly deletant, ∆44.047 Da and ∆23.081 Da is the difference between Leg_1 and Leg_2 or Leg_3, respectively. ∆15.995 Da corresponds to the addition of an oxygen atom on Met 12.

**Figure 5:**
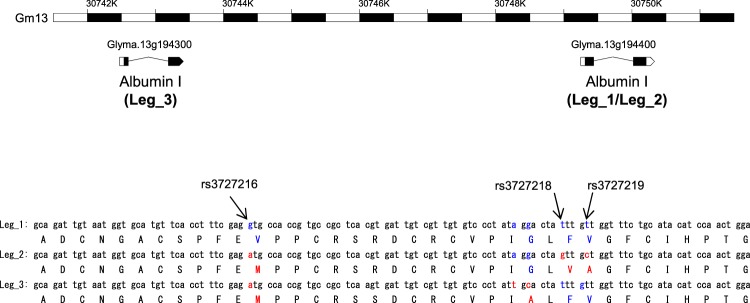
Location of *leginsulin* gene in soybean genome. Positions of SNPs are indicated by arrow in cDNA and peptide sequence of leginsulin variants.

It is suggested that the variation of the amino acid sequence in leginsulin is strongly associated with the difference in bioactivities of leginsulin homologues^2,3,9,10^. Additionally, potential leginsulin variants were observed in *aomarukun* (#5) sample (Fig. 3). Therefore, we further investigated the varietal distribution of leginsulin variants in 11 soybean cultivars (12 samples). As expected by PCA in Fig. 2C, Leg_1 (36 + 37) was detected in green-colored soybean seeds from *echigomidori*, *kiyomidori*, and *akitasanaodaizu* and also yellow-colored *enrei* (Fig. 4). In other samples, three MS ion peaks that differed from Leg_1_37 by ∆44.047 Da (RT 6.9 min), ∆28.052 Da (RT6.7 min) and/or ∆23.081 Da (RT 7.8 min) were detected. Additionally, in green soybean *aomarukun* seeds, the MS ion peaks shifted with ∆57.016 Da, which corresponded to the C-terminal Gly deletion, were also observed. Sequence analysis of cDNA obtained from *aomarukun* revealed two variants (GeneBank# AJ574791.1 and JF718836.1), both of which are translated into albumin 1 protein (UniPlot Q7XZC3 and G3FGW7), and encoding Leg_2 (∆44.055 Da) and Leg_3 (∆23.081 Da), respectively (Fig. 5). The sequence analysis of the cDNA clones of *albumin 1* derived from *aomarukun* showed that the occurrence ratio of Leg_2: Leg_3 was 9: 1, likely indicating Leg_3 is a minor variant in the cultivar. The soybean database (Soybase, http://www.soybase.org/) revealed that non-synonymous SNPs including rs3727216, rs3727218, and rs3727219 are located on Leg_1/Leg_2 coding region corresponding to the amino acid substitution, V12M, F28V, and V29A, indicating that Leg_1 and Leg_2 are homologous to each other (Fig. 5). Moreover, the genes of the precursor protein (albumin 1) of Leg_1/Leg_2 and Leg_3 are tandemly located within Gm13:30749261.0.30750344 (Glyma.13g194400) and Gm13:30742527.0.30743438 (Glyma.13g194300), respectively (Fig. 5). Therefore, genes encoding Leg/1Leg_2 and Leg_3 are defined as paralogous genes.

Additionally, LC-MS/MS analysis revealed that the sifted peak with ∆28.062 from Leg_1_37 was identified as Met12 oxidation of Leg_2_37 (Fig. 4B). The difference in theoretical molecular masses between reduced and oxidized forms of Leg_2_37 was ∆15.995, which is equivalent to that of a single oxygen atom. An MS ion peak shift with ∆15.995 corresponding to the Met12 oxidation was also observed in Leg_2_36 as well as Leg_3 (Fig. 4). Methionine residue is the most easily oxidized amino acid in peptides, while Leg_1 has no methionine residue in its amino-acid sequence. Met12-oxidized Leg_2_37(ox) was detected as a dominant form in immature *edamame* seeds (sample#11), but not in mature *edamame* (sample#12). We have previously revealed that the Leg_1 in 70% EtOH extract of *echigomidori* was oxidized by singlet oxygen derived from chlorophyll-mediated photosynthesization^11^. Therefore, Met12 oxidation of Leg_2 and Leg_3 is probably an artifact produced during sample preparation (Fig. 4).

### Expression levels of leginsulin variants

**Figure 6:**
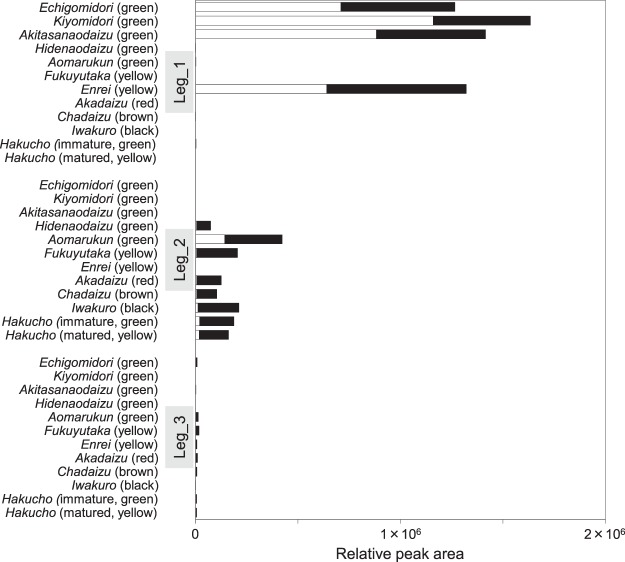
Varietal distribution of leginsulin homologues in 11 soybeans cultivars (12 samples). Peak areas of leginsulins analyzed by LC-MS were summarized (mean values, n = 3 in each sample). Open and closed bars represent leginsulins consisting of 36 (Gly deletant) and 37 amino acids, respectively. Peak areas detected as oxidized form were combined to a single column.

Expression levels of Leg_1 were markedly higher than Leg_2 and Leg_3 (Fig. 6). The C-terminal Gly deletants were detected in Leg_1 expressing cultivars and Leg_2 expressing *aomarukun*. Interestingly, *aomarukun*, a cultivar expressing Leg_2_36 abundantly, showed a level of Leg_2 (36 + 37) that was ~2-fold higher than other Leg_2-expressing cultivars. The levels of Leg_2 were not significantly changed during the seed maturation in *edamame hakucho* (sample#11 and 12). Expression levels of Leg_3 were very weak, results that were parallel to the cDNA frequency that appeared in sequence analysis.

### Insulin-like activities of leginsulin

**Figure 7:**
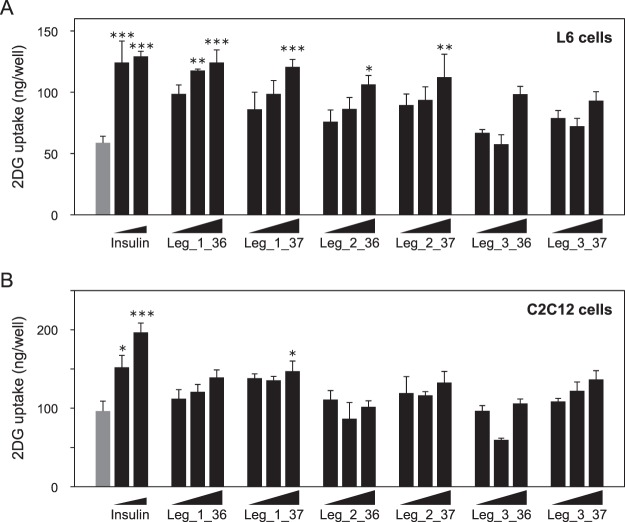
Myotube-like differentiated rat L6 (**A**) and mouse C2C12 (**B**) skeletal cells were treated with insulin (0, 0.1, 1.0 µM) or recombinant leginsulin variants (0.1, 1.0, 10 µM) for 24 h. Insulin-like activities were evaluated by the amount of 2-deoxyglucose uptake. *p < 0.05, **p < 0.01, ***p < 0.001, when compared with control (0 µM insulin; gray bar) by one-way ANOVA with post hoc test (Dunnet).

**Figure 8:**
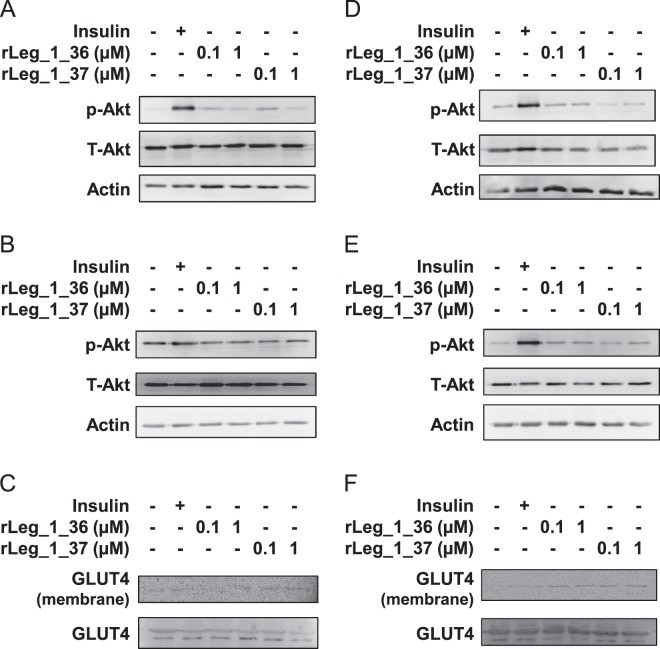
Myotube-like differentiated rat L6 (**A**–**C**) and mouse C2C12 (**D**–**F**) skeletal cells were treated with 1.0 µM insulin or recombinant leginsulin variants (rLeg_1_36 or rLeg_1_37) at indicated concentrations for 15 min (**A**,**D**) or 6 h (**B**,**C**,**E**,**F**). Immunoblot analyses of the total cell lysate and membrane fraction were performed using specific antibodies for phosphorylated-Akt (p-Akt), total-Akt (T-Akt), actin, and GLUT4.

To evaluate the insulin-like activities of leginsulins found in this study, TRX-His-tag fused recombinant leginsulins (rLegs) were prepared and exposed to myotube-like differentiated rat L6 and mouse C2C12 cells, and 2DG uptake was examined (Fig. 7). We confirmed that bovine insulin, which was used as a positive control, dose-dependently enhanced 2DG uptake in both cell lines. In rat L6 cells, the 2DG uptake was significantly up-regulated by rLeg_1 and rLeg_2 in a dose-dependent manner. In contrast, only the high-dose of rLeg_1_37 significantly induced 2DG uptake in mouse C2C12 cells. However, rLeg_3 did not stimulate 2DG uptake in both cell lines. Additionally, to examine molecular mechanisms of leginsulin-induced 2DG uptake, Akt signaling and GLUT4 expression were evaluated (Fig. 8). Akt activations were observed in both cell lines after treatment with rLeg_1_36 and rLeg_1_37, in which rapid response (15 min) were detected in L6 cells (Fig. 8A), but the sustained activation (6 h) were observed in C2C12 cells (Fig. 8E). GLUT4 translocations to the plasma membrane were also detected both cell lines after treatment with rLeg_1_36 and rLeg_1_37 (Fig. 8C,F). These results suggest that leginsulin-induced 2DG uptake would be mediated by insulin signaling pathway.

## Discussion

We investigated the varietal distribution of leginsulin in 11 soybean cultivars and detected three homologous leginsulins, Leg_1, Leg_2 and Leg_3, and their C-terminal Gly deletants, in which Leg_2 and Leg_3 were novel variants identified for the first time in this study. Amounts of Leg_1 observed in some green-colored soybeans and a yellow-colored *enrei* were markedly higher than Leg_2 content in other Leg_1-absent cultivars. The genotype of Leg_1 or Leg_2 was not related to the color of soybean seedcoat and cotyledon. Leg_3 was a novel paralogous peptide with a small expression level in the seeds.

Soybean leginsulin has an ability to stimulate the carrot callus redifferentiation and proliferation^5^, therefore is thought to be one of the plant peptide hormones. However the physiological role of leginsulin in soybean is still unidentified. If leginsulin is playing a role as peptide hormone in soybean, the growth and differentiation would be affected by the varietal gene sequence and expression. Soybean cultivars used in this study are varieties for foods. Therefore, if leginsulin variants are not related to their taste or harvest volume, it is not possible to observe the biased distribution of variants.

Although there is no sequence similarity between the leginsulin and insulin or insulin-like growth factors, topological similarity of the active residues of the Leg_1_37 and animal insulin was demonstrated^7^. In this study, we found that Leg_1_37 significantly induced insulin-like activities evaluated by 2DG uptake assay and immunoblotting in both myotube-like differentiated L6 and C2C12 cells (Figs 7 and 8). Leg_2 at high concentration induced 2DG uptake in differentiated L6 cells, but not in C2C12 cells. Therefore, the order of insulin-like activities of the leginsulin variants seems to be Leg_1 > Leg_2 > Leg_3. To trigger insulin-like activity, rLeg probably interacts with a cell surface molecule such as a “receptor”, because rLeg is a 21 kDa protein. Since delayed Akt activations were observed in C2C12 cells, some cell-type specificity including the expression level or the affinity to ligand could be involved with the response to leginsulin. Additionally, Leg_1 receptor Bg and animal insulin receptor have a structural resemblance, and Bg self-phosphorylation can be stimulated by animal insulin^6,12^. We demonstrated here that rLeg_1_37 activated Akt and induced the translocation of GLUT4 to the plasma membrane, which are located on the downstream of insulin signaling. Although further analyses are required, Leg_1 and Leg_2 possibly induced insulin-like activities via insulin receptors.

Amino acid substitutions from Leg_1 to Leg_2 were observed at position 12 (V12M), 28 (F28V) and 29 (V29A) (Fig. 5). Likewise, substitutions from Leg_1 to Leg_3 were at 12 (V12M), 18 (R18S) and 26 (G26A). The dissociation constant (*K*_*D*_) for Ala-substitution variants of rLeg to Bg had been previously determined^7,12^. These studies revealed that I25, F28, V29, F31, and I33 are located within the densely surface of rLeg and they are critical for the affinity to Bg. The affinity of V29A variant with Bg was drastically decreased with the greatest (116-fold) increase of *K*_*D*_^7^. In addition, *K*_*D*_ values of F28A, V12A, and R18A also increased 38.9-, 11.4-, and 5.69-fold, respectively^7^. Therefore, amino acid substitutions observed in Leg variants including V29A, F28V, V12M and R18S play critical roles in the interaction with and activation of not only Bg, but also insulin receptors. These results support the stronger insulin-like activities of Leg_1 variants.

The post-translation mechanism in the processing of albumin 1 protein to produce leginsulin remains unclear. Moreover, it is also unknown how the production of C-terminal Gly deletant is determined. Because, C-terminal Gly deletant was observed only in Leg_1-expressing cultivars (#1, 2, 3, and 7) and Leg_2-expressing *aomarukun* (#5). Many bioactive peptides including neuropeptide and hormones possessing Gly on their C-terminal are amidated by peptidylglycine α-amidating monooxygenase (PAM) to induce their full biological activities^13^. Additionally, glargine (GKR) is a longer-lasting human insulin analogue, which C-terminal Gly-extended form (GKR-Gly) is amidated by PAM to form GKR-NH_2_, inducing advantageous hypoglycemic activities in streptozotocin-injected type-I diabetic model Wistar rat^14^. Although, amidated forms of leginsulin (∆58.01 Da) were not observed in this study, insulin-like activities of Gly deletant were slightly weakened. Two forms consist of 37 and 36 amino acid, respectively, were not distinguished by the reverse phase column separation. Their chemical properties, therefore, seem to be similar, although C-terminal Gly deletion could be associated with the affinity with insulin receptor.

In the current study, we prepared TRX-His-tag fused rLeg, which were then used in *in vitro* analyses to evaluate insulin-like activities in mammal cells. Therefore, it will be necessary to prepare a large scale of each Leg variant by using tag-removal and purification, and apply them to animal experiments to evaluate their absorption rate and stabilities in *in vivo*. Lu and co-workers reported that at least some parts of orally injected PA1bB (called aglycin in their study) were absorbed and detected in mice plasma by HPLC-UV, although it was not quantified^2^. Interestingly, they have also demonstrated that PA1bB promotes glucose uptake not only in C2C12 cells, but also in insulin resistant C2C12 cells prepared by the chronic presence of insulin. Although the molecular mechanism underlying how PA1bB promotes glucose uptake in insulin resistant C2C12 cells has not been revealed, PA1bB seems to be enhancing insulin sensitivity via up-regulation of insulin receptors and insulin receptor substrate followed by the activation of insulin-induced GLUT4 transition in the skeletal muscle of diabetic model mice. Therefore, further studies are required to examine the bioavailabilities of Leg variants and their mechanisms of anti-diabetic activities.

Because insulin induced anti-inflammatory activities through the NF-kB inhibition in LPS-stimulated alveolar macrophage, leginsulin might be potentially equivalent^15^. Additionally, oral administration of insulin ameliorated the pancreatic cellular inflammatory process in non-obese diabetic mice without metabolic effect on blood glucose levels^16^. Soybeans are including other functional food factors such as isoflavone and saponins (Fig. 2). These phytochemicals are capable of inducing several health-beneficial effects for not only diabetes but also other inflammation-related diseases^17^. Therefore, the synergistic effects of Leg and soybean phytochemicals must be evaluated in future studies. Moreover, leginsulin is highly contained in Asian soybean cultivars^18,19^. Therefore, ingestion of cultivars that have high levels of accumulated Leg_1 would be favorable to the prevention of inflammatory related disorders.

## Materials and Methods

### Soybean samples

Soybean cultivars (appearances shown in Fig. 2A) including green soybean (cultivars; *echigomidori*, *kiyomidori*, *akitasanaodaizu*, *hidenaodaizu*, and *aomarukun*), yellow soybean (*fukuyutaka* and *enrei*), red (*akadaizu*), brown (*chadaizu*), and black (*iwakuro*) were obtained or gifted from a local store. *Edamame* (*hakucho*) plant was also purchased in a local store and cultivated to obtain immature and mature *edamame*. Crushed soybean powder (30 g) was delipidated in 30 mL of *n*-hexane at 175 rpm for 15.5 h in dark. After the filtration, the residue was dried *in vacuo* for 8 h, then extracted with 25 mL of 70% EtOH at 175 rpm for 17.5 h in the dark. After the filtration, 70% EtOH extract was evaporated and freeze-dried. The freeze-dried samples were stored at −20 °C until use.

### LC-MS

The freeze-dried samples dissolved in 70% EtOH (1 mg/ml) were injected ultra-performance liquid chromatography and time-of-flight mass spectrometry (UPLC-TOF-MS). The UPLC-TOF-MS system consisted of an Acquity™ UPLC (Waters, Milford, MA) and a MicroQTOF II mass spectrometer (Bruker, Bremen, Germany). UPLC separation was performed with an Acquity UPLC™ BEH C18 column (1.7 μm, 50 mm × 2.1 mm i.d., Waters) at 40 °C, using solvent A (0.1% formic acid in water) and solvent B (MeCN containing 0.1% formic acid). Samples were eluted from the column using a linear gradient of 5% solvent B from 0 to 2 min to 90% solvent B at 13–16 min. The flow rate of the mobile phase was 0.4 ml/min. The TOF-MS was operated in the positive ion mode using an electrospray ionization source (ESI +). The detector conditions were as follows: capillary voltage at 4500 V, nebulizer at 1.8 bar, drying gas flow at 10 l/min, drying gas temperature 200 °C, and the mass range between 50–2500 *m/z*. All analyses were performed using a low concentration tuning mix (Agilent technologies, Palo Alto, CA) to calibrate accurate mass. MS peak data from UPLC-TOF-MS analyses were subjected to Compass Data Analysis (Bruker) and Signpost (Reifycs, Tokyo Japan) for peak detection and integration. The value of raw data (peak area) were transformed to logarithm, and average values of experimental replicate (n = 3 in each groups) were divided with standard deviation to obtain Z-score, which were then subjected to principal component analysis (PCA) performed by Mass Profiler Professional software (Agilent Technology).

### Plasmid construction for leginsulin cDNA sequencing and bacterial expression

cDNA including soybean *albumin 1* gene were prepared RT reaction of total RNA, which was extracted from the freeze-dried sample using TRIzol and SuperScript III reverse transcriptase™ (Invitrogen), and were then amplified by PCR using the following primer set: 5′-CTT GCT CCT ATG GCT GTC TT-3′ and 5′-GCA GTA GAT GCA TCA TAA GC-3′. The amplified oligonucleotides were cloned into the *EcoRV* and *HindIII* restriction site of pET32a(+) vector. Plasmids were transformed into DH5α competent cells, amplified by miniprep, then purified using a QIAprep spin miniprep kit (QIAGEN). Sequence analyses were performed using a BigDye terminator v3.1 cycle sequencing kit by ABI 3100 Avant. The cDNAs encoding Leg_1, 2, 3 on the sequenced plasmids were individually amplified using the following primers to construct the bacterial expression systems: N-terminal primer: 5′-AAC CAT GGA AAG CAG ATT GTA ATG GTG CAT-3′ and C-terminal primer: 5′-AAG AAT TCT TAT TAT CCA GTT GGA TGT ATG CAG AA-3′ for leginsulin consisting of 37 amino acids or 5′-AAG AAT TCT TAT TAT CAA GTT GGA TGT ATG CAG AA-3′ for the deletion of C-teminal Gly construct. The amplified DNA were cloned into the *NcoI* and *EcoRI* restriction site of the pET32a(+) vector and transformed into Origami (DE3) competent cells. Transformed cells were grown at 37 °C in 100 ml of LB medium, containing 50 µg/ml ampicillin, until the OD_600_ value reached 0.6. After addition of ITPG (final 0.1 mM), cells were incubated at 37 °C for 6 h and harvested by centrifugation. Cells were suspended in 50 mM phosphate buffer (pH 7.0) containing 300 mM NaCl, then sonicated. The soluble fraction was clarified by centrifugation and applied to the TALON column (Clontech) to purify TRX-His-tag fused leginsulin (rLeg).

### Glucose uptake assay

Insulin-like activities of rLeg were evaluated by a 2-deoxyglucose (2DG) uptake assay according to previously described methods^20,21^. Briefly, rat L6 and mouse C2C12 skeletal muscle cells were differentiated into myotubes by cultured in 2% FBS/DMEM for 5 days, then exposed to recombinant leginsulin or bovine insulin in 2% FBS/DMEM for 4 h. The washed myotubes with Krebs–Ringer–phosphate–Hepes (KRPH) buffer (pH 7.4, 20 mM Hepes, 5 mM KH_2_PO_4_, 1 mM MgSO_4_, 1 mM CaCl_2_, 136 mM NaCl, 4.7 mM KCl) containing 0.1% BSA were incubated with KRPH buffer containing 1 mM 2DG and 0.1% BSA for 1 h at 37 °C in 5% CO_2_. The cells were lysed by the addition of 0.1M NaOH, then subjected to freeze-thaw treatment, and incubated at 85 °C for 50 min. After neutralization by 0.1 M HCl, assay cocktail (50 mM TEA, pH 8.1; 50 mM KCl; 0.5 mM MgCl_2_; 0.02% BSA; 670 µM ATP; 0.12 µM NADP^+^; 25 µM resazurin sodium salt; 5.5 units/ml hexokinase; 16 units/ml G6PDH; and 1 unit/ml diaphorase) was added. After the incubation at 37 °C for 1 h, fluorescence intensity (Ex 544 nm, Em 590 nm) was measured. The amount of 2DG uptake was determined by a standard curve generated by 2DG6P standard solutions. Statistical analyses were performed with EZR (Saitama Medical Center, Jichi Medical University), which is a graphical user interface for R (The R Foundation for Statistical Computing). One-way ANOVA followed by Dunnet test was used for the comparisons between control and other test groups. Differences were considered significant at *p* < 0.05.

### Western Blotting

Total cell lysates were prepared by lysis buffer (50 mM Tris-HCl, pH 8.0; 150 mM NaCl, 0.1% SDS, 0.5% deoxycholate, and 1% Triton X-100). On the other hands, membrane fraction was obtained according to previous report^22^. In brief, cells were harvested with buffer A (50 mM Tris-HCl, pH 8.0, and 0.5 mM dithiothreitol) containing 0.1% NP-40), and disrupted by passing through a 25-gauge needle 10 times. The homogenate centrifuged at 1,000 × *g* for 10 min at 4 °C, and the precipitate was suspended in NP-40-free buffer A and recentrifuged at 1,000 × *g* for 10 min at 4 °C. The precipitate was suspended again in buffer A containing 1.0% (v/v) NP-40 and incubated for 1 h at 4 °C. After the incubation, each homogenate was centrifuged at 16,000 × *g* for 2 0 min at 4 °C. The supernatant was collected and stored as the plasma membrane fraction at -80 °C until use. Samples were separated by SDS-PAGE, then blotted onto PVDF membrane. Antibodies for the detection of specific protein were anti-AKT (#9272, Cell Signaling Technology), anti-Phospho-AKT (Ser473) (#4058, Cell Signaling Technology), anti-GLUT4 (07–1404, Merck), and anti-Actin (A2066, Sigma-Aldorich). The bands visualized using LuminoGraph I (Atto Co., Tokyo, Japan).

## Electronic supplementary material

Supplementary Information
