# Supplementary material for: Identification of soybean peptide leginsulin variants in different cultivars and their insulin-like activities
Source: Sci Rep. 2018 Nov 15;8:16847. doi: 10.1038/s41598-018-35331-5 (PMC6237985; doi:10.1038/s41598-018-35331-5)
Supplement: Supplementary file 1 — Supplementary Information [file 41598_2018_35331_MOESM1_ESM.pdf]

Supplementary information

Identification of soybean peptide leginsulin variants in different cultivars and their insulin-like activities

Tsutomu Hashidume[1,2], Taiken Sakano[1], Ayaka Mochizuki[2], Keisuke Ito[1,2], Sohei Ito[1,2], Yasuaki Kawarasaki[1,2], Noriyuki Miyoshi[1,2]

From the [1]Graduate School of Integrated Pharmaceutical & Nutritional Sciences,

[2]School of Food & Nutritional Sciences, University of Shizuoka, Shizuoka 422-8526, Japan

Full images of IB (Figure 8)

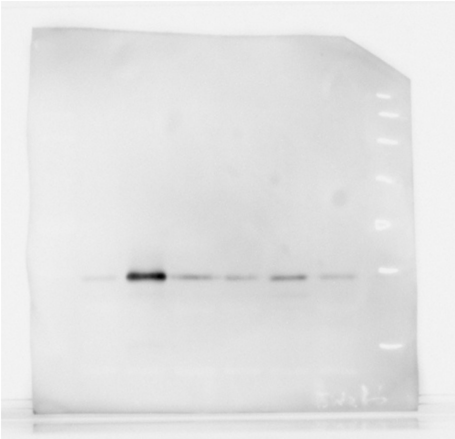

Cell; L6
Time; 15 min
Antibody; p-AKT

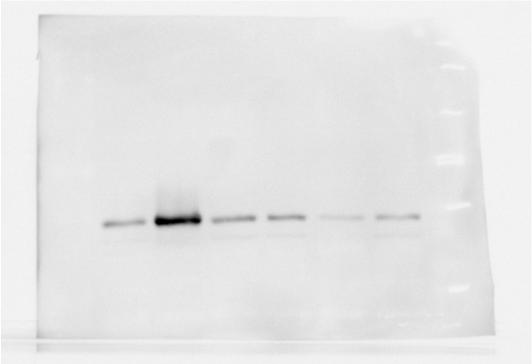

Cell; C2C12
Time; 15 min
Antibody; p-AKT

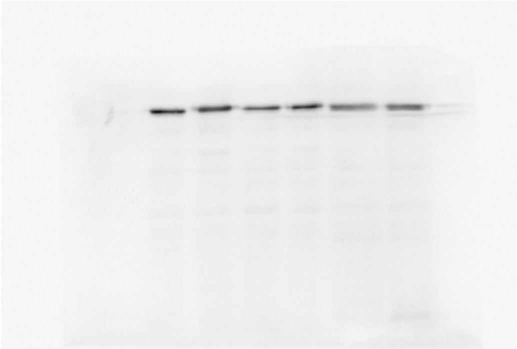

Cell; L6
Time; 15 min
Antibody; total-AKT

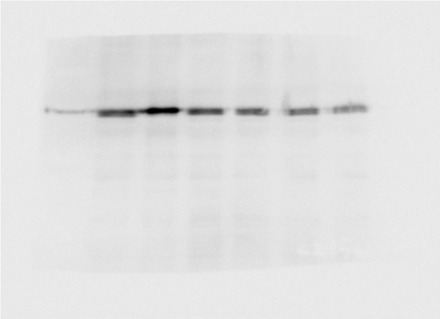

Cell; C2C12
Time; 15 min
Antibody; total-AKT

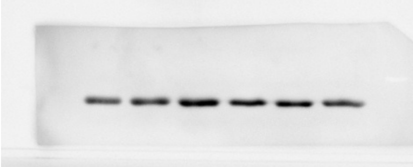

Cell; L6
Time; 15 min
Antibody; actin

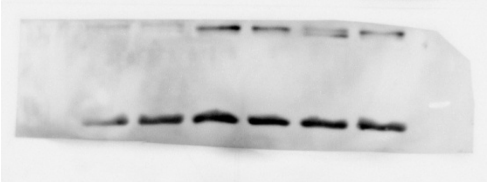

Cell; C2C12
Time; 15 min
Antibody; actin

Full images of IB (Figure 8)

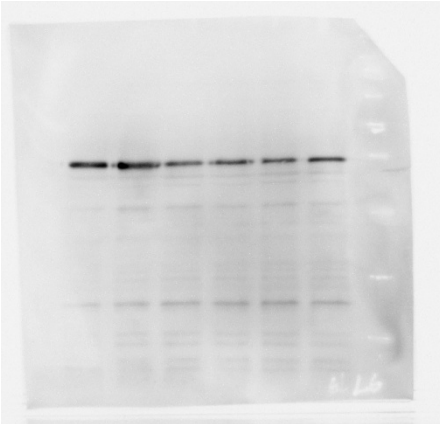

Cell; L6
Time; 6 h
Antibody; p-AKT

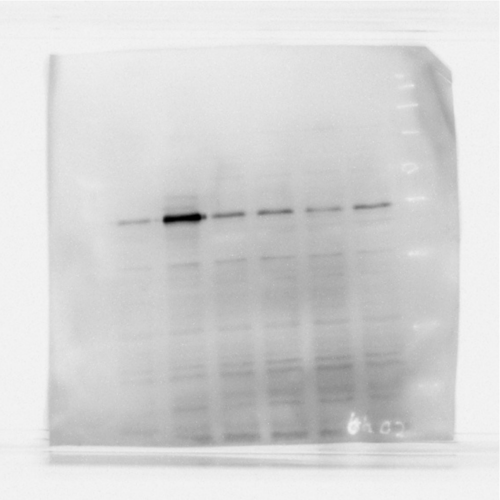

Cell; C2C12
Time; 6 h
Antibody; p-AKT

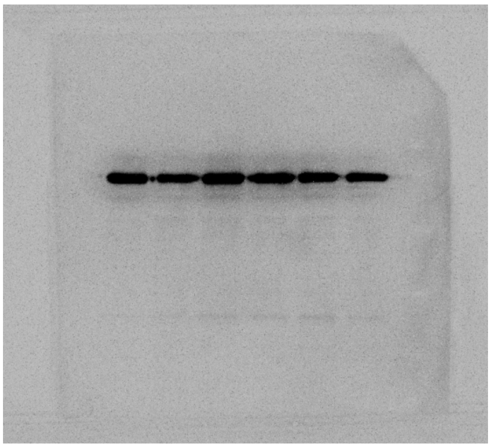

Cell; L6
Time; 6 h
Antibody; total-AKT

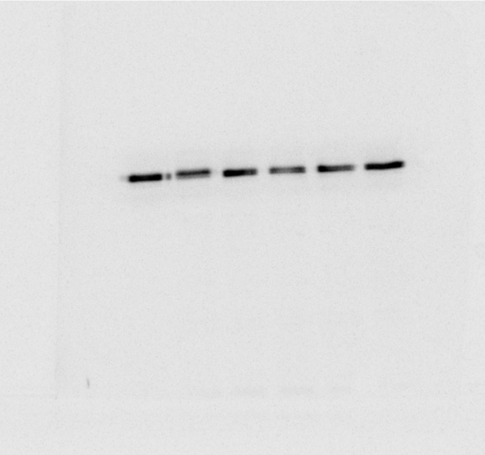

Cell; C2C12
Time; 6 h
Antibody; total-AKT

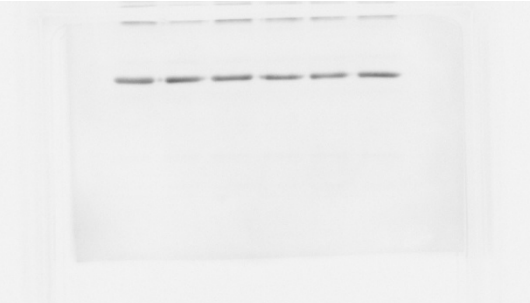

Cell; L6
Time; 6 h
Antibody; actin

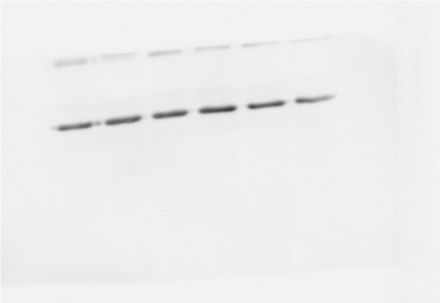

Cell; C2C12
Time; 6 h
Antibody; actin

Full images of IB (Figure 8)

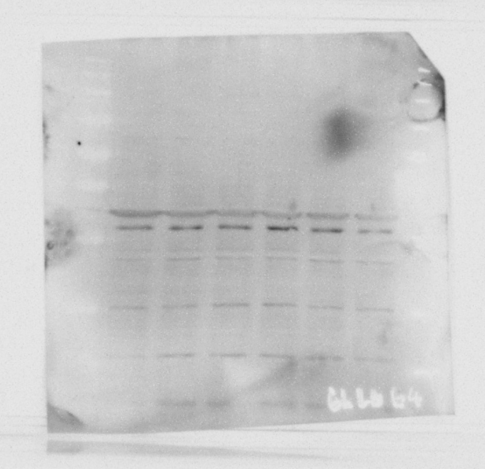

Cell; L6
Time; 6 h
Antibody; GLUT4

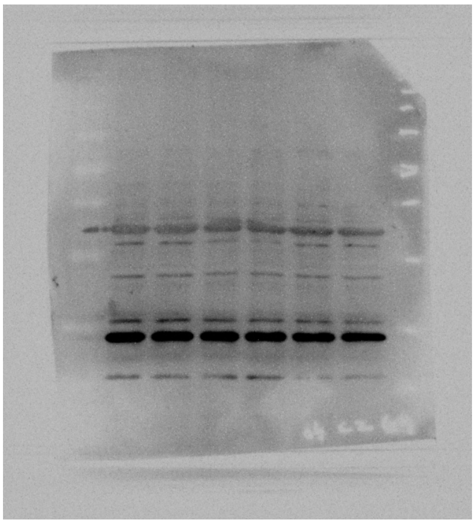

Cell; C2C12
Time; 6 h
Antibody; GLUT4

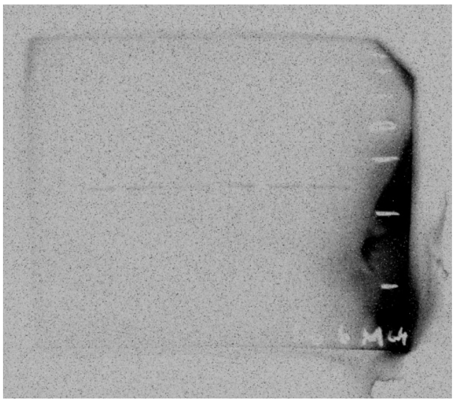

Cell; L6 membrane fraction
Time; 6 h
Antibody; GLUT4

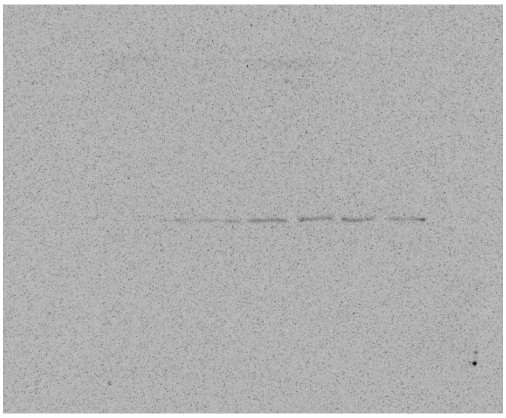

Cell; C2C12 membrane fraction
Time; 6 h
Antibody; GLUT4
